# A root–soil association index reveals life‐history strategies of arbuscular mycorrhizal fungi

**DOI:** 10.1111/nph.71338

**Published:** 2026-06-04

**Authors:** Iñaki Odriozola, Felix Wesener, Petr Baldrian, Florian Barbi, Lukas Bell‐Dereske, Karel Klem, Lenka Meszárošová, Otso Ovaskainen, Jakub Skřivánek, Tomáš Větrovský, Zuzana Kolaříková, Petr Kohout

**Affiliations:** ^1^ Institute of Microbiology of the Czech Academy of Sciences Vídeňská 1083 142 00 Prague Czechia; ^2^ Global Change Research Institute of the Czech Academy of Sciences Bělidla 986/4a 603 00 Brno Czechia; ^3^ Department of Biological and Environmental Science University of Jyväskylä P.O. Box 35 FI‐40014 Jyväskylä Finland; ^4^ Faculty of Science Charles University Albertov 6 128 00 Prague Czechia; ^5^ Institute of Botany of the Czech Academy of Sciences Zámek 1 252 43 Průhonice Czechia

**Keywords:** biomass allocation, community ecology, ecological traits, GlobalFungi, Glomeromycota, Glomeromycotina, mycelia

## Abstract

- Arbuscular mycorrhizal (AM) fungal taxa differ in their life‐history strategies, including their preferential association with intraradical vs extraradical compartments.
- We introduce the *arbuscular mycorrhizal root*–*soil association index* (AMF‐RSI), a novel quantitative index describing the relative association of AM fungi with root vs soil compartments, corresponding to the previously proposed rhizophilic and edaphophilic AM fungi. The AMF‐RSI was calculated from metabarcoding data compiled in the GlobalAMFungi database using Hierarchical Modeling of Species Communities and was subsequently applied to assess shifts in AM fungal community traits driven by soil disturbance caused by tillage.
- We assessed 301 AM fungal taxa and found that AMF‐RSI was strongly conserved at the genus level. While the majority of AM fungal taxa from families Archaeosporaceae, Diversisporaceae and Paraglomeraceae exhibited preferential association with soil, taxa within Glomeraceae exhibited high variability in edaphophilic vs rhizophilic strategies. AMF‐RSI values predicted AM fungal community responses to soil disturbance, with rhizophilic taxa being more prevalent in tilled soils.
- The AMF‐RSI represents a new quantitative trait distinguishing preferential association of AM fungi with intraradical vs extraradical compartments (edaphophilic vs rhizophilic life‐history strategies). Applied at the community level, it provides a tool for characterizing shifts in AM fungal communities in response to environmental conditions.

## Graphical abstract

Phylogenetic tree of Glomeromycota, including all studied virtual taxa, based on the small subunit region. The bar plot in the external part of the phylogeny depicts the log‐transformed *arbuscular mycorrhizal root*–*soil association index*, with values > 0 indicating rhizophilic lifestyle, < 0 edaphophilic lifestyle, and close to 0 no preference. The color of the bars depicts the statistical support, which indicates the posterior support for the rhizophilic or edaphophilic strategy of each virtual taxon.

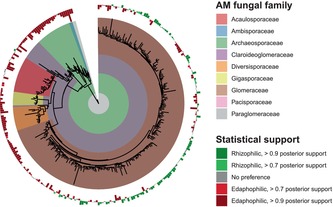

## Introduction

Arbuscular mycorrhizal (AM) fungi, belonging to the phylum Glomeromycota (Schüßler *et al*., 2001; Tedersoo *et al*., 2018), are important mutualistic symbionts of > 70% of plant species, including many agricultural crops (Brundrett & Tedersoo, 2018). As obligate biotrophs, they assist plants in acquiring mineral nutrients from soil and protecting them against pathogens (Smith & Read, 2008). Consequently, AM fungi play key roles in plant establishment and productivity, stress mitigation (Smith & Read, 2008; Kakouridis *et al*., 2022), and soil carbon sequestration (Hawkins *et al*., 2023). Despite their importance, only *c*. 370 species have been formally described, with many more yet undiscovered (Corradi *et al*., 2025). Although AM fungi are known to differ in their effects on host plants (Kiers *et al*., 2011) and ecosystem processes such as soil aggregation and nutrient retention (Qiu *et al*., 2022), their cryptic, biotrophic lifestyle, and strong environmental context dependence makes detailed understanding of species‐level ecology methodologically challenging (Antunes *et al*., 2025). As a result, we still lack predictive frameworks linking AM fungal species composition to plant performance, vegetation dynamics, and soil properties.

Functional traits determine how species respond to biotic and abiotic variation and thus influence their adaptability to environmental conditions (Moretti & Legg, 2009). Traits that mediate species' responses to environmental change – the so‐called response traits – provide mechanistic and predictive insights into community assembly and ecosystem dynamics (Suding & Goldstein, 2008; Cadotte *et al*., 2015). Accordingly, trait‐based approaches often yield more ecologically relevant perspectives than analyses based solely on species identities (McGill *et al*., 2006; Cadotte *et al*., 2011). Over the past decades, experimental studies have documented striking variation among AM fungal taxa in structural characteristics, including root colonization extent and morphology (Hart & Reader, 2002; Klironomos & Hart, 2002), hyphal length, density, and architecture (Avio *et al*., 2006; Powell *et al*., 2009), frequency of hyphal fusions (Voets *et al*., 2006), and spore traits such as size, color, and abundance (Bever *et al*., 2001; Oehl *et al*., 2009). These characteristics seem to be conserved at the species level (Powell *et al*., 2009; Koch *et al*., 2017), although substantial intraspecific variation in extraradical biomass production has also been observed (Sun *et al*., 2024).

Since the AM fungal thallus consists of two main compartments: extraradical mycelia in soil, which primarily captures nutrients, and intraradical mycelia within host roots, in which mineral nutrients are exchanged for plant‐derived carbon (Keymer *et al*., 2017; Luginbuehl *et al*., 2017), the differences in relative share of these compartments should identify life‐history strategies and be linked to ecological functions. Taxa allocating more biomass to soil mycelia (‘edaphophilic’ fungi; Weber *et al*., 2019) are considered efficient phosphorus foragers, whereas those allocating more biomass to roots (‘rhizophilic’ fungi; Weber *et al*., 2019) are expected to be resistant to mechanical disturbances of soil, such as tillage (Powell *et al*., 2009; Chagnon *et al*., 2013), and to provide better protection for plant roots against pathogens (Sikes, 2010). Thus, quantifying the relative investment of AM fungi into extraradical vs intraradical structures may provide a useful functional trait for understanding variation in AM fungal life‐history strategies and their ecological consequences.

Edaphophilic vs rhizophilic life‐history strategies have previously been applied as a functional trait to better explain the role of AM fungi in soil carbon sequestration (Horsch *et al*., 2023), to understand the effect of ecosystem degradation on AM fungal communities (Dong *et al*., 2025), and to predict responses of AM fungal communities to nitrogen addition (Han *et al*., 2020). However, most of these studies relied on broad categorizations at the family level – for example, Glomeraceae are often described as rhizophilic fast growers, whereas Gigasporaceae and Diversisporaceae are considered edaphophilic slow growers with low root colonization. Yet evidence for family‐level conservatism is based on a limited number of isolates (Powell *et al*., 2009), methodologically problematic estimates of AM fungal biomass (Sharma & Buyer, 2015; Olson & Lekberg, 2022), and a narrow gradient of environmental conditions. Moreover, the relative investment of AM fungi into extraradical vs intraradical structures likely represents a continuum rather than discrete categories. This highlights the need to characterize AM fungal life‐history strategies at finer taxonomic resolution, across a broader range of taxa and to use noncategorical variables. Quantitative evaluation of the relative investment of AM fungi into extraradical vs intraradical structures therefore represents a significant step toward assessing differences in the ecological functions of AM fungal communities in relation to various environmental conditions.

Here, we aimed to describe the root‐ vs soil‐associated life‐history strategy of AM fungi across diverse taxa by introducing the AM fungal root–soil association index (AMF‐RSI), a quantitative trait‐like metric describing the relative investment of AM fungal taxa into extraradical vs intraradical structures. To calculate AMF‐RSI, we modeled the relative abundance distribution of AM fungal taxa by Hierarchical Modeling of Species Communities (Ovaskainen *et al*., 2017) and, for each taxon, we calculated the ratio of expected relative abundance in root and soil samples, while accounting for the effect of methodological, environmental, and spatial factors. For that, we used AM fungal metabarcoding data compiled in the GlobalAMFungi database (Větrovský *et al*., 2023). Given that GlobalAMFungi contains mostly root and soil samples originating from different spatial locations, we applied spatial filtering strategies at contrasting spatial resolutions and we accounted for environmental biases within the modeling process. Additionally, we conducted sensitivity analyses between filtering thresholds to assess the robustness of our results. The relative abundance ratio of taxa (based on the metabarcoding amplicon sequencing data compiled in the GlobalAMFungi database) between root and soil samples was used as a proxy for the relative investment of AM fungal taxa into extraradical vs intraradical structures. We further tested the phylogenetic conservation of AMF‐RSI. Then, we validated AMF‐RSI by examining AM fungal community data from colocated root and soil samples in two independent studies not used to compute the index, as well as by assessing its correlation with neutral lipid fatty acid (NLFA)‐based biomass estimations of experimentally grown AM fungal isolates. Lastly, we tested the relevance of AMF‐RSI for the characterization of AM fungal community assembly.

## Materials and Methods

### Identification of arbuscular mycorrhizal fungal life‐history strategy

#### Distribution data of arbuscular mycorrhizal fungi

To determine the differences of the relative investment of AM fungal taxa into extraradical vs intraradical structures, we utilized the data on the relative abundances of these taxa in soil and root samples available in the GlobalAMFungi database, which contains the most comprehensive collection of AM fungal metabarcoding data available to date (Větrovský *et al*., 2023). The relative abundance of each taxon in each root and soil sample is defined as the proportion of sequences assigned to that taxon relative to the total number of sequences in the sample. For the purpose of our analysis, we derived data about AM fungal taxa occurrence from the small subunit (SSU) dataset. Raw sequences from 76 SSU studies (representing the 1st release of the GlobalAMFungi database) were subjected to sequence quality check, trimming of the sequences to the V4 region of the SSU, and assignment of the sequences to Virtual Taxa (VT), based on the most recent release of type sequences of VT from the Maarj*AM* database from 5 June 2019 (Öpik *et al*., 2010). We constructed two AM fungal community matrices using different sequence similarity thresholds. In the first (‘97% dataset’), sequences were assigned to a VT when query coverage was ≥ 98% and sequence similarity was ≥ 97%. In the second (‘99% dataset’), sequences were assigned to a VT when query coverage was ≥ 98% and sequence similarity was ≥ 99%. AM fungal VT are defined based on phylogenetic analyses of SSU rRNA region and roughly correspond to AM fungal species or higher taxonomical units (see Öpik *et al*., 2014). Although other barcoding regions may provide better resolution for some lineages within AM fungi (Schlaeppi *et al*., 2016), molecular identification based on VT is traditionally used for AM fungal metabarcoding studies using the SSU region.

Because of an uneven distribution of GlobalAMFungi database samples among biomes, we constrained our analyses to forest and grassland, the two most thoroughly sampled biomes. Our final dataset comprised 4969 samples originating from 47 studies, covering information about the occurrences of 338 AM fungal VT. All rhizosphere (140), topsoil (3), and soil (2353) samples were considered as soil samples, resulting in 2496 samples. By contrast, 2473 samples were derived from host plant roots. The dataset encompasses a very broad diversity of host plants: In total, root samples were obtained from > 250 plant species. The vast majority of samples were collected from single‐host species samples, whereas pooled whole‐community root samples accounted for only 6.8% of all root samples. Among the single‐host samples, only one species was relatively overrepresented, namely *Plantago lanceolata* (14.2%); all other plant species each contributed < 3% of the root samples.

All samples were associated with exact GPS coordinates, based on the information from the original publications and were assigned to continents. We obtained a set of key environmental variables from global databases, representing broad gradients of climate and fundamental soil properties. Climatic variables, mean annual temperature (MAT), and mean annual precipitation (MAP) were retrieved for each sample, based on its geographical location from the global CHELSA database (Karger *et al*., 2017), representing the data from the period 1981–2010. Soil characteristics were retrieved for each sample, based on its geographic location from the global SoilGrids database (Hengl *et al*., 2017). We used soil pH and soil organic C (SOC) from 0 to 5 cm of soil depth. Additionally, we classified the samples into forest and grassland biomes to account for major differences in vegetation context.

**Fig. 1:**
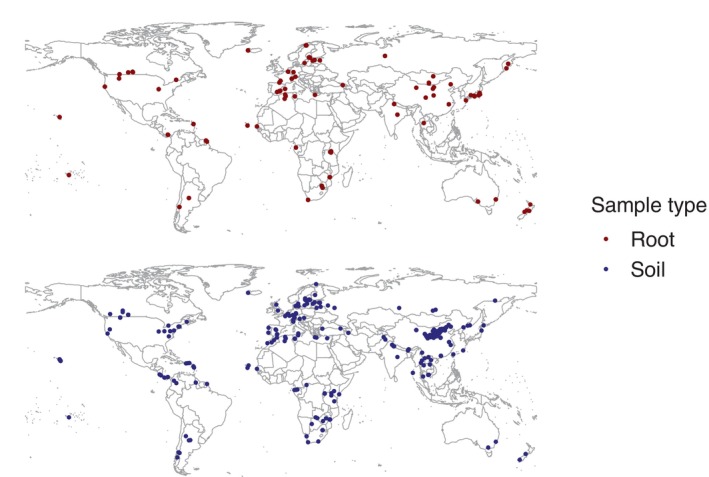
Distribution of root (blue) and soil (red) samples from the GlobalAMFungi database and included in the 1000‐km dataset (i.e. includes 1729 soil samples with a reference root sample within 1000‐km distance and 2718 root samples with a reference soil sample within 1000‐km distance). These data were used for modeling of root vs soil association of arbuscular mycorrhizal fungal taxa.

From each of the two datasets defined by 97% and 99% sequence similarity thresholds, two datasets were generated using alternative spatial distance thresholds for filtering: 1000 and 100 km. Within each dataset, we analyzed all VT occurring in at least five samples. The 1000‐km dataset included root samples that had a reference soil sample within a distance of 1000 km, and soil samples that had a reference root sample within the same distance. Both soil and root samples showed balanced global distribution (Fig. 1). The more restrictive 100‐km dataset included root samples that had a reference soil sample within a distance of 100 km, and soil samples that had a reference root sample within the same distance. Spatial filtering ensured comparable spatial distributions and environmental conditions between root and soil samples, particularly in the 100‐km dataset (Fig. 1; Supporting Information Figs S1, S2).

Therefore, in total, four datasets were used to compute the index: the 1000‐km distance and 97% similarity dataset, consisting of 4447 samples (2718 from roots and 1729 from soil) and 301 AMF VT; the 100‐km distance and 97% similarity dataset, containing 1927 samples (1423 from roots and 504 from soil) and 268 AMF VT; the 1000‐km distance and 99% similarity dataset, containing 3819 samples (2231 from roots and 1588 from soil) and 211 VT; and the 100‐km distance and 99% similarity, containing 1480 samples (1023 from roots and 457 from soil) and 173 VT. Comparisons across datasets differing in sequence similarity (97% and 99%) and spatial distance (1000 and 100 km) thresholds served as a sensitivity analysis assessing the robustness of our results to the chosen taxonomic and spatial filtering resolutions.

#### Statistical analysis

We modeled the differential relative abundance between sample types (root vs soil) of individual AM fungal taxa with the Hmsc framework (Hmsc model hereafter), which belongs to the class of Joint Species Distribution Models (Warton *et al*., 2015), through the R package hmsc (Tikhonov *et al*., 2020). Then, we defined the AMF‐RSI as the ratio between the expected relative abundance of each AM fungal VT between root and soil samples. Given the nature of metabarcoding data, AMF‐RSI should be considered an indicator of intraradical vs extraradical allocation preferences for individual AM fungal VTs, rather than a measure of absolute biomass. This index was subsequently used in a case study (will be discussed later) to identify its applicability to assessment of traits of whole AM fungal communities. We specifically focused on the community trait response of AM fungal communities subject to mechanical disturbance. The complete code to reproduce the application of the Hmsc models and posterior calculation of the AMF‐RSI is available in a Zenodo repository at doi: 10.5281/zenodo.18325103.

As the data were zero‐inflated, we applied a hurdle model (zero‐altered model) (see Ovaskainen & Abrego, 2020, for more details about Hmsc models, including how hurdle models can be used to model zero‐inflated data). This type of model consists of two parts, one modeling the presence–absence of species and the other modeling abundance conditional on presence. To fit the first model, we transformed all nonzero values in the dataset into one to create a presence–absence matrix. We applied a binomial model with a probit link function to each AM fungal VT. The second model looked at abundances conditional on presences (scaled to mean zero and unit variance). We transformed zeros to missing values and kept all nonzero values; we then fitted the log‐normal model. Then, the two components of the model were fitted consecutively. As fixed explanatory variables, we included the categorical factor substrate type (with two levels: roots and soil), as well as the categorical factor environment classification (with two levels: forest and grassland) and the continuous variables pH, SOC, MAT, and MAP, and log‐transformed sequencing depth, which accounted for differences in sequencing depth between samples. Quadratic transformations of all continuous variables except sequencing depth were also included to allow nonlinear relationships. Additionally, we included continent as a random effect to further account for possible spatial biases in the datasets.

To examine whether the responses of the AM fungal VT to fixed effects showed a phylogenetic signal, we included in the analysis a phylogenetic correlation matrix C among the species (for more details, see Ovaskainen *et al*., 2017). We downloaded VT‐type sequences from the 2019 MaarjAM release (trimmed between NS31‐AML2 primers; primer sequences were removed) for 326 VTx present in the GlobalAMFungi database. Sequence alignment was performed in Mafft v. 7.222 (Katoh & Standley, 2013) using the Q‐INS‐i strategy, which accounts for rRNA secondary structure. Poorly aligned positions and divergent regions were removed from the alignment using the software Gblocks v.0.91b (Castresana, 2000) under less stringent conditions. Maximum likelihood analyses were conducted in Iq‐Tree v. 2.1.3 (Nguyen *et al*., 2015) with automated model selection (‐m TESTNEW) via Modelfinder (Kalyaanamoorthy *et al*., 2017), using 1000 SH‐aLRT bootstrap replicates (‐alrt 1000) and 1000 ultrafast bootstrap replicates (‐bb 1000). Based on the Akaike Information Criterion, the selected model was TIM2 + F + R5. The final alignment included 326 sequences comprising 439 nucleotide sites, of which 109 were constant and 211 were parsimony‐informative. The phylogenetic signal is measured using the parameter ρ, which takes values from 0 to 1, a value of 0 meaning no phylogenetic signal in the responses to environmental variables (beta parameters of Hmsc), and a value of 1 meaning completely phylogenetically structured responses.

We fitted the models using default prior assumptions and estimated model parameters by sampling from their posterior distributions using Markov Chain Monte Carlo (MCMC) methods, a standard approach for Bayesian inference. Specifically, we ran four independent MCMC chains, each with 3750 iterations. The first 1250 iterations of each chain were discarded to allow the algorithm to stabilize (burn‐in), and every tenth iteration was retained thereafter, resulting in 250 posterior samples per chain and 1000 samples in total. Model convergence was assessed using the potential scale reduction factor, which evaluates whether the independent chains converged to the same solution (Tikhonov *et al*., 2020). Convergence diagnostics for the beta parameters, which quantify the responses of VT to fixed effects, are provided in Figs S2 and S3.

To assign AMF‐RSI to individual AM fungal VT, we used the previously described hurdle models to generate predicted abundances of each VT in roots and in soil and divided the values to create a ratio that indicates the strength of the association of a VT with roots. Specifically, we defined the expected abundance of a VT in a specific habitat as the product of the posterior median probability of presence obtained from the binomial submodel and the expected abundance obtained by exptransforming the posterior median prediction from the lognormal submodel. Then, the ratio between the expected abundance in roots and soil can be interpreted as the number of times the abundance of a VT is higher in roots than in soil. For instance, a ratio of two indicates that the expected relative abundance is double in roots compared to soil; a ratio of one indicates equal relative abundances in both habitats, and a ratio of 0.5 indicates half relative abundance in roots. In the few cases where a VT was entirely absent from one of the habitats, the abundance component is undefined for that compartment. In these cases, the AMF‐BAI is informed exclusively by the presence–absence submodel, and the resulting ratio can be interpreted analogously as the number of times a VT is more (or less) likely to occur in roots than in soil. Thus, whether informed by both submodels or by the presence–absence component alone, the AMF‐RSI consistently represents a relative measure of root vs soil association. When making the predictions for the habitats, we kept the nonfocal explanatory variable environment classification at the reference level and the environmental covariates and sequencing depth in their mean values, thus making the comparisons under standardized environmental conditions and sequencing efforts. The standardized sequencing effort made expected abundances relative.

To measure the phylogenetic scale at which the predicted ratio was structured, we built a phylogenetic correlogram linking the VT AMF‐RSI and the patristic phylogenetic distance between VT using the function phyloCorrelogram() from the R package phylosignal (Keck *et al*., 2016). Patristic distance is the evolutionary distance between two taxa, measured as the sum of the branch lengths separating them on a phylogenetic tree. Last, we calculated credible intervals of 90% and 70% and used them to classify AM fungal VT into five categories of AMF‐RSI for visualization: (1) strong evidence for root association: VT with a ratio value > 1 and 90% credible interval not overlapping 1; (2) moderate evidence for root association: a ratio value > 1 and 70% credible interval not overlapping 1; (3) neutral VT: 70% credible interval overlapping 1; (4) moderate evidence for soil association: a ratio value < 1 and 70% credible interval not overlapping 1; and (5) strong evidence for soil association: a ratio value < 1 and 90% credible interval not overlapping 1. We emphasize that the AMF‐RSI is a continuous quantity, and the credible intervals should primarily be interpreted as measures of uncertainty around each VT‐specific estimate rather than as strict thresholds for hypothesis testing.

### Validation of the arbuscular mycorrhizal fungal root–soil association index

#### Metabarcoding approach

To identify replicability of the AMF‐RSI, we selected two studies (Faghihinia *et al*., 2021; Ma *et al*., 2025) based on the following criteria: (1) AM fungal taxa were identified using the SSU rRNA gene region. (2) AM fungal communities were assessed in paired root and soil samples. (3) The study has not been included in the GlobalAMFungi data used for calculation of the AMF‐RSI. From each study, we extracted the AM fungal community matrix, sample metadata, and AM fungal taxonomy. Then, we assigned the log‐transformed AMF‐RSI value from the 1000‐km dataset to each AM fungal VT to calculate the community‐weighted means (CWM) of AMF‐RSI, based on raw sequence counts. Before calculating CWMs, we applied the Hellinger transformation to the AM fungal VT relative abundances to downweight the influence of common species and upweight the influence of the rare ones in the analysis.

To test the null hypothesis of no difference in CWM of AMF‐RSI between AM fungal communities in root and soil samples from the same locations, we fitted linear mixed models for each study using the lmer function from lme4 R package (Bates *et al*., 2015) and used lmertest R package (Kuznetsova *et al*., 2017) to compute the *P*‐values. For Faghihinia *et al*. (2021), fixed effects included grass species (*Stipa grandis*, *Leymus chinense*), substrate (root, soil), and grazing intensity (0–9 sheep ha^−1^), including their interactions, with random effects for sample pairs (root–soil samples) and transects. For Ma *et al*. (2025), the variance associated with the random effect (describing the randomized block design) was estimated to be near zero; therefore, we proceeded with a linear model instead after removing correlated covariates.

#### Experimental approach

To further validate the AMF‐RSI, we performed a pot experiment assessing the intra‐ and extraradical biomass of eight AM fungal isolates belonging to seven different species. Namely we used two isolates of *Claroideoglomus claroideum* (BEG23 and AZ2250) and one isolate of *Diversispora toruosa* (JA306A), *Funneliformis mosseae* (BEG95), *Gigaspora margarita* (BEG34), *Paraglomus brasilianum* (BR105), *Rhizophagus irregularis* (PH5), and *Septoglomus deserticola* (NC302A). As a host plant, we used *Plantago lanceolata* cv LIBOR. AM fungi were supplied as single species inocula from long‐term cultures, with one plant per pot and six replicate pots per AM fungal isolate. Pots were filled with a layer of autoclaved expanded clay and washed quartz sand. A liquid inoculum (5 ml) containing spores, hyphae, and colonized root fragments was applied below 5‐d‐old *P. lanceolata* seedlings to ensure direct root–inoculum contact. All plants received the same balanced nutrient solution supporting growth of AM fungi and plants and were also watered regularly. After 60 d, we harvested the experiment. Whole root systems were washed free of substrate and weighed fresh. A subsample of roots (0.5 g of fresh weight) and a subsample of homogenized substrate (5 g of fresh weight) were collected for NLFA analysis, lyophilized, and stored at −20°C until processing. The remaining roots were dried at 60°C to constant mass to determine dry weight, and total root dry weight was calculated by scaling with whole fresh root system.

AM fungal biomass was estimated using the NLFA biomarker 16:1ω5, which is widely used as a proxy for AM fungal biomass (Olson & Lekberg, 2022). Lipids from root and substrate samples were extracted, subjected to mild alkaline methanolysis, and analyzed by gas chromatography mass spectrometry. NLFA 16:1ω5 concentrations were converted to absolute amounts per root system and per pot of substrate by multiplying by the weight of total dry roots or by the weight of a reference pot filled with substrate. Average root : soil ratio of the absolute NLFA values per isolate were then correlated with the AMF‐RSI values calculated based on the Hmsc models.

### Application of the arbuscular mycorrhizal fungal root–soil association index to arbuscular mycorrhizal fungal community ecology

To identify applicability of the AMF‐RSI in AM fungal ecology, we evaluated its predictive utility on AM fungal communities affected by soil physical disturbance using an agricultural field experiment with differences in tillage.

#### Experimental design

The samples were collected at two experimental agricultural fields, Babicka (49°40.4′N, 16°27.5′E; 460 m above sea level (asl)) and Vetrolam (49°39.9′N, 16°28.4′E; 475 m asl) near the municipality of Banín, Czechia. At each field, four experimental blocks (100 × 5 m) were established in autumn 2019, separated by 3.3‐m‐wide grass strips. Each block was split lengthwise, with one‐half conventionally tilled (CT) and the other under nontilled (NT) management, separated by a 50‐ to 75‐cm aisle (depending on the crop). NT management also included the cultivation of species‐rich cover crops with > 10 components in the period after the harvest of the main crop, which was terminated later by frost (when the following crop was sown in spring), or by sowing the main crop in autumn (in the case of winter wheat). The crop rotation was the same on all plots: 2020 spring barley, 2021 maize, 2022 spring triticale, and 2023 winter wheat. Within each block and tilling treatment, amendment treatments were applied (addition of different bio residues). All plots were sown with triticale in 2022 and with winter wheat (*Triticum aestivum* var. *aestivum*) in October 2023 using a no‐till seeder (Väderstad Rapid 300).

Soil and root samples were collected from Babicka on 1 November 2023 and from Vetrolam on 9 November 2023. Sampling was conducted using a handheld sampling drill (Ø 5 cm) to a depth of 15 cm. Three subsamples were taken from each plot representing the combination of replication and treatment and put immediately into a mobile freezer with −20°C and transported to the laboratory within 6 h for further processing. In the laboratory, subsamples were pooled and sieved through a 2‐mm mesh, after which roots were separated from each sample. Both soil and root samples were then stored at −80°C before being freeze‐dried for further analyses.

#### Metabarcoding of arbuscular mycorrhizal fungal communities

Total genomic DNA was extracted in duplicates using 250 mg of freeze‐dried soil samples, following a modified Miller method (Sagova‐Mareckova *et al*., 2008). DNA extracts were cleaned using the Geneclean Turbo Kit (MP Biomedicals, Solon, OH, USA) and pooled before subsequent PCRs. AM fungal communities were identified using sequencing of the variable regions of V4/V5 regions of the SSU amplified by AM fungal‐specific primers NS31 (Simon *et al*., 1992)/AML2 (Lee *et al*., 2008). PCR conditions, amplicon library preparation, sequencing, and bioinformatic methods were the same as previously described in Kohout *et al*. (2024). Operational Taxonomic Units, constructed at a 97% similarity level, were classified to one of the AM fungal VT only when the sequence coverage ≥ 98% and sequence similarity ≥ 97% against representative sequences of the Maarj*AM* database from 5 June 2019 (Öpik *et al*., 2010).

#### Statistical analysis

We assigned the log‐transformed AMF‐RSI values to each detected AM fungal VT and calculated the CWM of AMF‐RSI based on Hellinger‐transformed AM fungal VT relative abundances for each sample. In this study, we aimed to focus on the tilling treatment; therefore, we averaged the response variables across the amendment treatments and analyzed the experiment as a randomized complete block design, replicated over two sites (Casella *et al*., 2008). To assess the effect of tillage, we employed mixed effects models using the lmer() function from the lme4 R package (Bates *et al*., 2015), and we used the lmertest R package (Kuznetsova *et al*., 2017) to compute the *P*‐values. We included categorical fixed effects for tillage (CT and NT), compartment (root and soil samples), and site (Babicka and Vetrolam), including interactions between these variables. We included block as a random effect. To ensure the robustness of our results, we verified that the conclusions remained quantitatively similar without the averaging over amendment treatments. We visually verified that the model residuals met the assumptions of homoscedasticity and normality.

## Results

### Root–soil association of arbuscular mycorrhizal fungal taxa

AMF‐RSI was calculated using four alternative datasets for Hmsc modeling, combining two alternative sequence similarity thresholds when mapping sequences to the AM fungal VT with two alternative spatial distance thresholds used to filter the data: 1000 and 100 km, such that only those root and soil samples were included in the analysis if complementary samples were within 1000 or 100 km, respectively. The four analyses yielded similar results in terms of the estimated median AMF‐RSI (Figs S4, S5). We then compared the uncertainty of the estimates across the four datasets. Notably, the dataset combining a 1000‐km distance and 97% sequence similarity – which modeled the largest number of VT and used the largest amount of data – yielded the predictions with the lowest uncertainty (Fig. S6). This indicates that the precision gained from reducing genetic noise (99% similarity) and spatial bias (100‐km spatial distance) may be overwhelmed by the loss of precision resulting from reduced sample size and spatial and environmental representativeness. Therefore, we present results hereafter based on the 1000‐km distance and 97% similarity dataset (Table S1), because it included a larger number of taxa and samples, while being consistent with the more conservative datasets. The results for the datasets with 100‐km distance and 97% similarity, 1000‐km distance and 99% similarity, and 100‐km distance and 99% similarity are summarized, respectively, in Tables S2–S4.

**Fig. 2:**
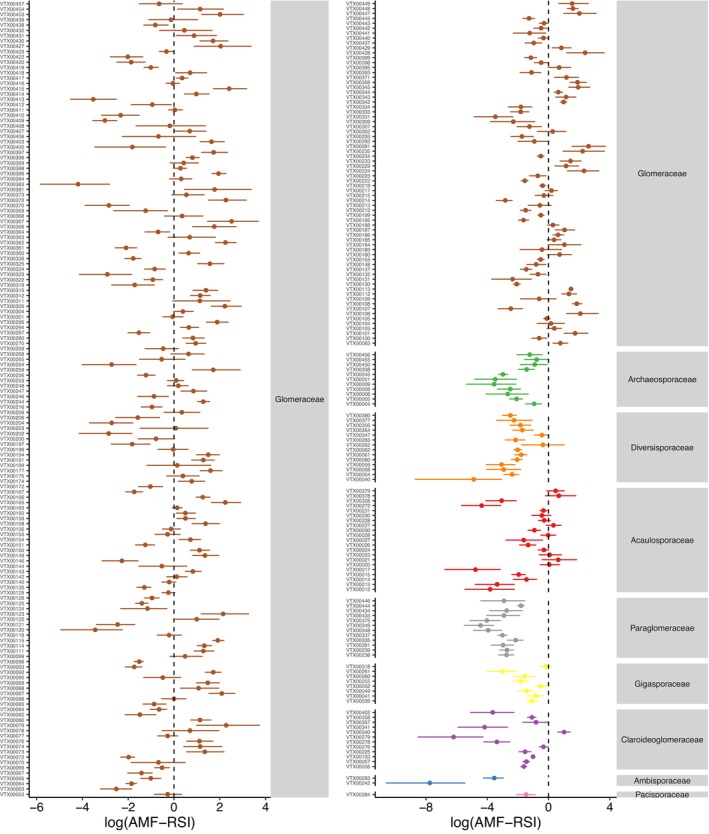
Averages and credible intervals (CI) of log‐transformed arbuscular mycorrhizal fungal root–soil association index (AMF‐RSI) for the studied arbuscular mycorrhizal (AM) fungal Virtual Taxa. Forest plot representing the average values and 70% CI of the estimated ratio, indicative of rhizophilic vs edaphophilic lifestyle of different AM fungal taxa. The error bar crossing the dashed vertical line indicates a probable neutral distribution pattern (i.e. compartment association preference under a 70% posterior support threshold), whereas a bar to the left indicates significantly higher relative abundance in soil (i.e. edaphophilic lifestyle) and to the right indicates significantly higher relative abundance in roots (i.e. rhizophilic lifestyle).

Altogether, using the 1000‐km distance and 97% similarity dataset, we were able to assess AMF‐RSI for 301 AM fungal VT from nine major AM fungal families (Fig. 2). Among them, 82 VT showed significant affinity to root samples (AMF‐RSI > 1 based on 70% posterior statistical support), indicating their rhizophilic lifestyle, while 145 VT were preferentially found in soil, indicating their edaphophilic lifestyle. The remaining 74 VT did not show a clear preference for root nor soil samples.

**Fig. 3:**
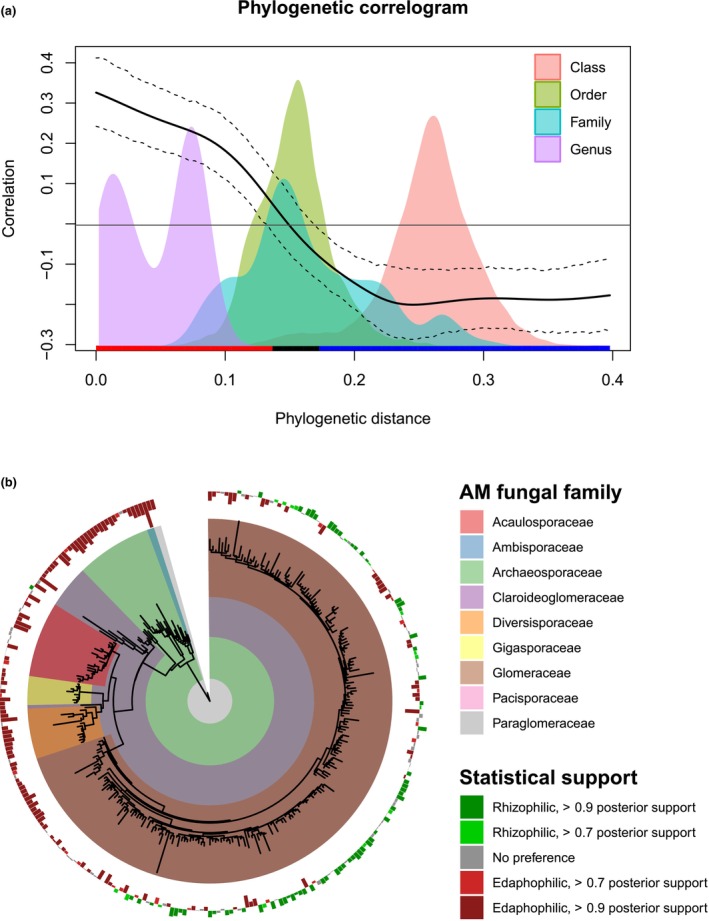
Phylogenetic structure of arbuscular mycorrhizal fungal root–soil association index (AMF‐RSI). (a) Phylogenetic correlogram showing the scale at which the AMF‐RSI was structured. Solid line indicates mean correlation value and dashed lines indicate 95% bootstrap confidence intervals. Colored densities encompass pairwise Patristic phylogenetic distances between Virtual Taxa from different taxonomic classifications. The horizontal color bar in *x*‐axis indicates positive phylogenetic correlation (red), no significant phylogenetic correlation (black) and negative phylogenetic correlation (blue). (b) Phylogenetic tree of Glomeromycota, including all studied virtual taxa, based on the small subunit region. The bar plot in the external part of the phylogeny depicts the log‐transformed AMF‐RSI, with values > 0 indicating rhizophilic lifestyle, < 0 edaphophilic lifestyle and close to 0 no preference. The color of the bars depicts the statistical support, which indicates the posterior support for the rhizophilic or edaphophilic strategy of each virtual taxa. > 0.9 support indicates that the 90% of the posterior credible interval obtained a ratio value larger or smaller than one; > 0.7 support indicates 70% CI obtained values larger or smaller than 1; 70% credible interval overlapping one was classified as no preference.

Both components of the Hmsc hurdle model (presence–absence and abundance conditional on presence) showed statistically supported phylogenetic signal (ρ = 0.73 with 90% CI (0.67, 0.79) for presence–absence component, and ρ = 0.68 with 90% CI (0.61, 0.75) for the abundance component, respectively) in species association with root vs soil samples. Accordingly, AMF‐RSI was strongly conserved on genus level, and partly on family and order levels, suggesting that closely related AM fungal VT share a similar compartment association strategy (Fig. 3a). Members of the families Ambisporaceae, Archaeosporaceae, Diversisporaceae, Gigasporaceae, Pacisporaceae, and Paraglomeraceae mostly showed edaphophilic lifestyle. Edaphophilic lifestyle also prevailed among VT from the families Acaulosporaceae and Claroideoglomeraceae, but not exclusively. By far the highest diversity of root–soil association strategies was found among the Glomeraceae, in which particularly the early branching lineages showed high affinity toward edaphophilic lifestyle, while rhizophilic lifestyle prevailed in late branching lineages (Fig. 3b). However, this pattern was not exclusive.

### Validation of the arbuscular mycorrhizal fungal root–soil association index

To identify replicability of AMF‐RSI, we selected two studies (Faghihinia *et al*., 2021; Ma *et al*., 2025), which aimed to identify composition of AM fungal communities in colocated root and soil samples. We were able to calculate CWM of the AMF‐RSI, based on 65 AM fungal VT (representing 94% of AM fungal sequences) identified by Ma *et al*. (2025) and 66 AM fungal VT (representing 69% of AM fungal sequences) identified by Faghihinia *et al*. (2021).

**Fig. 4:**
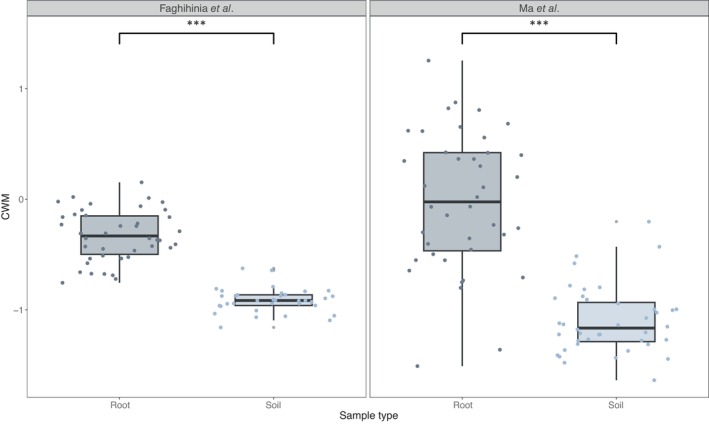
Community‐weighted mean (CWM) of arbuscular mycorrhizal fungal root–soil association index (AMF‐RSI) originating from the two selected studies. A higher value indicates arbuscular mycorrhizal (AM) fungal community with higher relative abundance of rhizophilic taxa. ***, *P* < 0.001.

Both of these datasets showed significantly higher CWM of AMF‐RSI in root than in soil samples, indicating higher relative abundance of rhizophilic AM fungi in roots than in soil samples (Faghihinia *et al*. (2021): df_nom_ = 1, df_den_ = 57.36, *F*‐value = 280.43, *P*‐value < 0.001; Ma *et al*. (2025): *F*‐value = 96.66, *P*‐value < 0.001; Fig. 4). This analysis, identifying a consistent pattern in AM fungal distribution between roots and soil across different ecosystems and experimental setups, indicates expectable distribution of AM fungal taxa according to the AMF‐RSI.

Congruently with the metabarcoding‐based validation of the AMF‐RSI, relative biomass allocation into extra‐ and intraradical mycelia (measured by NLFA concentration), of seven tested AM fungal species, positively correlated with the values of AMF‐RSI of the corresponding VT (Pearson's *r* = 0.81; *P*‐value = 0.01; Fig. S7). This result indicates that the values of AMF‐RSI might be cautiously interpreted as an indicator of different biomass allocation strategies of AM fungal taxa.

### Response of root–soil association of AM fungal communities to tillage

**Fig. 5:**
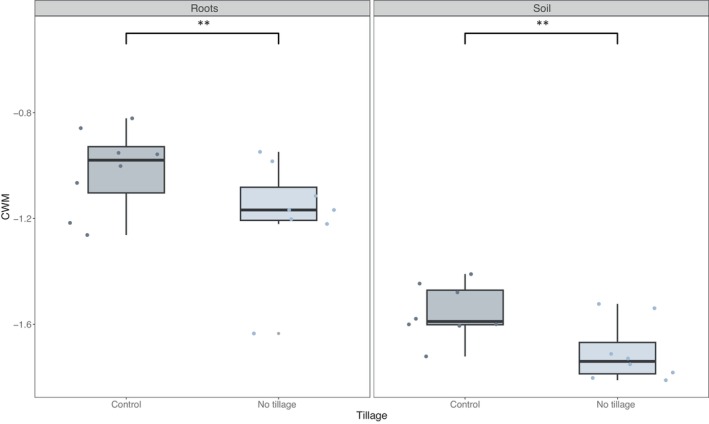
Community‐weighted mean (CWM) of arbuscular mycorrhizal fungal biomass allocation index (AMF‐RSI) between root and soil samples originating from two agricultural experimental study sites in Czechia. A higher value indicates arbuscular mycorrhizal (AM) fungal community with higher relative abundance of rhizophilic taxa. **, *P* < 0.01.

We calculated the CWM based on 65 AM fungal VT (representing 97% of AM fungal sequences). In agreement with the previously described results of validation of AMF‐RSI, our mixed modeling analysis revealed a significantly higher CWM of AMF‐RSI in roots than in soil for case study II (df_nom_ = 1, df_den_ = 18, *F*‐value = 110.78, *P*‐value < 0.001; Fig. 5), indicating higher relative abundance of rhizophilic species in root samples. We also found a significant positive effect of tillage on the CWM of AMF‐RSI (df_nom_ = 1, df_den_ = 18, *F*‐value = 9.31, *P*‐value = 0.007; Fig. 5), as it physically disrupts extraradical hyphae, shifting AM fungal communities toward higher relative dominance of rhizophilic taxa. There were no significant interactions; therefore, the effect of tillage was consistent across the two substrates (root and soil) and experimental sites, and the effect of environments was consistent across sites. Together, these results suggest a pervasive impact of tillage on AM fungal community composition, manifested as a shift toward rhizophilic taxa in ploughed soils – likely reflecting their greater tolerance of physical disturbance relative to edaphophilic taxa. Importantly, this disturbance‐shaped soil species pool appears to carry over into the root‐associated communities, in which rhizophilic AM fungi were even more prevalent at tilled than at NT sites.

## Discussion

This study introduces a novel quantitative life‐history trait – AMF‐RSI – that was hypothesized to exist (Weber *et al*., 2019) but never reported on species level of AM fungi. This trait describes preferential root‐ vs soil‐associated life‐history strategy of AM fungal taxa. The AMF‐RSI was calculated for individual AM fungal taxa based on their realized distribution data between root and soil habitats in the samples from real environment. Conceptually, this index corresponds to widely used indicator values in plant ecology (e.g. Ellenberg's indicator values), which are based on a classification of plant species according to the position of their realized ecological niche along an environmental gradient (Ellenberg *et al*., 1991; Tichý *et al*., 2023). Numerous studies have proven the merit of these observational‐based indices in ecological research (e.g. Kopecký *et al*., 2024; Wei *et al*., 2025). To calculate AMF‐RSI, we modeled the relative abundance distribution of AM fungal taxa by Hmsc (Ovaskainen *et al*., 2017) based on several thousands of soil and root samples, while accounting for the effect of technical, environmental and spatial factors. This resulted in ratio of expected relative abundances in root and soil habitat for each of 301 studied AM fungal taxa, together with posterior credible intervals, which we are now presenting as the new AMF‐RSI quantitative trait. This study also validated the AMF‐RSI by experimental assessment of intra‐ and extraradical biomass of selected AM fungal species, indicating that the AMF‐RSI might be considered as a good proxy of AM fungal biomass allocation strategies.

### Root–soil association strategies of AM fungi and the AMF‐RSI

Previous attempts aiming to better understand the root–soil association strategies (Weber *et al*., 2019) proposed the existence of three categories of AM fungi, those that preferentially invest their biomass to colonize roots (rhizophilic), soil (edaphophilic), or those with low biomass values (ancestral). Although this approach offered an interesting view of the functioning of AM symbioses (Phillips *et al*., 2019; Wang *et al*., 2023), there is no justifiable reason to expect that there are three distinct strategies for AM fungi biomass allocation between the root and soil environments. Plants, which are also foraging for different resources using different structures – roots for mineral nutrients and water, while shoots for carbon and light – neither exhibit just two distinct strategies of investment into above‐ vs belowground biomass, but instead show a continuum of root and shoot biomass ratio (Grime, 1977). Compared with the previous simplistic sorting of AM fungi into three functional groups, we argue that the AMF‐RSI provides a more accurate proxy for root–soil association strategies of AM fungal taxa, due to the quantitative nature of this trait.

The data about root–soil association strategies that were previously collected for just a handful of taxa used to be extrapolated to higher taxonomic levels and sorted AM fungi to functional groups on family level (Weber *et al*., 2019). Our results, based on SSU delimitation of AM fungi into the so‐called VT, showed phylogenetic conservation of the AMF‐RSI on genus rather than family level. This indicates that genera or species from a single family can differ in the root–soil association strategy, as we can clearly see on the example of the Glomeraceae family (Fig. 3). Therefore, we advocate for finer taxonomic resolution when attributing the root‐associated or soil‐associated life‐history strategy to AM fungal taxa. To do so, we provide the AMF‐RSI for 301 AM fungal VT. Although the delimitation of AM fungal VT based on SSU barcoding region does not enable resolution of all taxonomically described AM fungal species, as some closely related species were found to be indistinguishable (Schlaeppi *et al*., 2016), the fact that AMF‐RSI is phylogenetically conserved on genus level partly overcomes these limitations and indicates that the proposed index can be widely applied in different aspects of AM research.

The AMF‐RSI was calculated based on AM fungal metabarcoding data compiled in the GlobalAMFungi database. The GlobalAMFungi represents by far the largest available dataset of AM fungal taxa distributions (compiling AM fungal community data from 100 studies; Větrovský *et al*. (2023)), with *c*. 2500 samples coming from root and soil samples. Importantly, after applying the spatial filters, root and soil samples showed balanced global distribution (Fig. 1), which decreased the risk of potential biogeographical biases. Due to the fact that the GlobalAMFungi database is, similar to the GlobalFungi database (Větrovský *et al*., 2020), designed as a permanent effort that will be continuously updated by its creators and through the collaboration of the scientific community, we expect that a growing body of metabarcoding studies focused on AM fungal communities (Frew *et al*., 2025; Lutz *et al*., 2025) will enable more precise calculations of AMF‐RSI as well as the coverage of additional AM fungal VT. Capitalizing on the better taxonomical resolution of AM fungal taxa enabled by longer rRNA sequences (Kolaříková *et al*., 2021; Tedersoo *et al*., 2024), we plan to recalculate the AMF‐RSI using the data from the next version of the GlobalAMFungi database for taxonomically better resolved AM fungal species.

The obligate biotrophic nature of AM fungi presents a practical challenge for understanding the fungi themselves and biases interpretation of their ecology. It has been recently estimated that there are only *c*. 88 AM fungal species represented in culture collections world‐wide (Antunes *et al*., 2025). Although there has been an extensive effort to describe variation in AM fungal traits (Van Der Heijden & Scheublin, 2007; Chagnon *et al*., 2013; Behm & Kiers, 2014; Chaudhary *et al*., 2022; Antunes *et al*., 2025), traits were assessed for only a handful of easily culturable AM fungal taxa, due to practical limitations. In fact, the great majority of AM fungi are difficult to culture; moreover, the traits may vary with different host plants, which further complicates the generalization of trait‐based research across the whole Glomeromycota. On the contrary, molecular methods have the potential to provide insights into AM fungal ecology in various ecosystems around the globe across the entire diversity of Glomeromycota, including unculturable taxa (Větrovský *et al*., 2023) without the need of challenging cultivation experiments. By leveraging global AM fungal metabarcoding data, the AMF‐RSI provides a quantitative framework for describing root–soil association strategies across entire AM fungal diversity.

### Methodological limitations

Although this approach enabled us to utilize a massive dataset, it has some potential limitations that should be considered when interpreting the results. While AM fungal life‐history theories often focus on differential investment of AM fungal taxa into intra‐ and extraradical biomass, the biological relevance of this trait remains largely unclear. Camenzind *et al*. (2024) recently advocated for an increased focus on variation among structures foraging for the same element, that is within intra‐ and extraradical structures, than differential investment between them. As in case of plant ecology, in which the uptake of different elements (carbon vs mineral nutrients) is spatially decoupled as in case of AM fungi, numerous plant species, particularly those with stress tolerant life strategy or some of the long‐living, slow‐growing trees generally exhibit low plasticity in biomass allocation patterns, as their growth and morphology are evolutionarily constrained by adaptations to persistently stressful environments (Reich *et al*., 1997; Poorter *et al*., 2012; Freschet *et al*., 2013). Similarly, AM fungal taxa can differ in their plasticity in biomass allocation patterns, which can reflect certain outcomes depending on the combination of host plant and fungal species (Johnson *et al*., 1997; Chaudhary *et al*., 2022) or local environmental conditions, such as soil texture (Rillig & Steinberg, 2002). One could hypothesize that AM fungi with high plasticity can invest more into soil biomass (corresponding to low AMF‐RSI) when interacting with host plants with coarse roots or growing in soil with low nutrient levels, while they would invest more to intraradical biomass when interacting with plants with fine roots or growing in soil with high nutrient levels. In case of the proposed AMF‐RSI, we assume that taxa with high plasticity of biomass allocation patterns would probably show wide posterior credible intervals, while those with fixed biomass allocation ratios would have narrow posterior probability intervals. Importantly, intraspecific trait variability is not exclusive to the AMF‐RSI, but a general challenge for trait‐based ecological studies (Bolnick *et al*., 2011; de Bello *et al*., 2025). Future research may target the plasticity of RSI in AM fungi.

The AMF‐RSI was calculated based on AM fungal metabarcoding data compiled in the GlobalAMFungi database (Větrovský *et al*., 2023). While all metabarcoding data, gathered in the GlobalAMFungi database, were obtained using AM fungal‐specific primers and all raw sequencing data were processed using the standardized bioinformatic methods, the individual studies could have differed in their methodological approaches (e.g. amount of material used for DNA isolation, molecular kits, PCR‐associated biases, and sequencing platform), which could have potentially introduced methodological bias to the dataset that was used for AMF‐RSI calculation. However, these biases are likely not jeopardizing conclusions derived from our analyses but rather introduce additional noise over the natural biological patterns. Studies compiled in the GlobalAMFungi database originated from different environmental, geographical conditions, and host plant species, which brings another level of challenges associated with the aims to identify AMF‐RSI of individual AM fungal taxa. This challenge is particularly apparent when comparing our approach with experimental studies of AM fungal traits, in which environmental conditions can be controlled but may not capture real‐world environmental complexity (Antunes *et al*., 2025). While controlled experiments can limit the effects of environmental variability, our approach – inferring species‐level ecology through species distribution modeling – must explicitly account for substantial environmental and geographical heterogeneity in the data. To address this, all models were fitted using prefiltered data and incorporated environmental and spatial characteristics, which should enhance the robustness of the calculated index. Host plant identity is another potential source of bias. Because the dataset includes root samples from hundreds of host species (with low replication for most hosts), we could not explicitly incorporate host identity into AMF‐RSI calculation. However, AMF‐RSI estimates were highly consistent between the two spatial filtering schemes (1000 km vs 100 km), despite the large difference in sample sizes (4447 vs 1927, respectively), suggesting that host‐driven bias is likely modest for most VTs, although it cannot be fully excluded, especially for rare taxa. As additional root‐community datasets accumulate across a wider range of host plants, it will become possible to quantify host effects more directly and refine AMF‐RSI where needed. Nevertheless, by leveraging environmental samples, our approach at least partly embraces the complexity of natural communities and may therefore provide complementary, and in some respects more ecologically realistic, insight into life‐history strategies of AM fungal taxa.

### AMF‐RSI across Glomeromycota

Our study provides the first comprehensive assessment of the relative investment of AM fungi into extra‐ vs intraradical structures in the whole Glomeromycota, covering > 300 taxa across nine families. Most importantly, compared with previous efforts that assessed preferential biomass allocation on family level (e.g. Powell *et al*., 2009; Chagnon *et al*., 2013; Weber *et al*., 2019), we provide AMF‐RSI on biologically more relevant species level. Previously, Glomeraceae and Claroideoglomeraceae were considered as rhizophilic (investing more biomass to root than soil), while Gigasporaceae and Diversisporaceae were considered as edaphophilic (investing more biomass to soil than root). Our results support the prevailing edaphophilic strategy among Gigasporaceae, Paraglomeraceae, and Diversisporaceae species. We also identified a strong prevalence of edaphophilic strategies in the Archaeosporaceae family. This family was previously considered to form low biomass in soil as well as in roots (Weber *et al*., 2019). Although our methodological approach does not provide information about absolute values of AM fungal biomass, the results clearly show that the majority of Archaeosporaceae taxa preferentially associate with soil rather than roots. Other families, such as Claroideoglomeraceae and Acaulosporaceae, did not show such a consistent pattern in AMF‐RSI, although the edaphophilic strategy was prevailing.

Importantly, our study showed diverse ecological strategies of root–soil association within the family Glomeraceae, which was previously considered rhizophilic (Weber *et al*., 2019). Our data provide strong evidence that numerous lineages, particularly most early branching lineages, within the Glomeraceae family should be considered as edaphophilic (Fig. 3). This indicates that treating AM fungal species belonging to the same family as a uniform unit might result in too simplified a view on AM fungal ecology, especially in the case of the largest AMF family Glomeraceae. We also found that the AMF‐RSI is more conserved at the genus than family level, which is particularly obvious in Glomeraceae.

AM fungal preferential investment between extra‐ and intraradical phases has often been linked to ecological functions. Edaphophilic taxa were considered efficient phosphorus foragers, whereas rhizophilic species are expected to play a role in pathogen protection of the host plants (Chagnon *et al*., 2013). Based on high soil mycelium production, Gigasporaceae would be expected to outperform Glomeraceae in nutrient uptake (Maherali & Klironomos, 2007). A former meta‐analysis (Yang *et al*., 2017) suggested that, on average, fungi of the family Glomeraceae were better at acquiring P and reducing pathogen growth compared to other AM fungal families. Conflicts between the theoretical expectation and experimental evidence that rhizophilic Glomeraceae species are less suited for mineral nutrient uptake compared with the edaphophilic Gigasporaceae species might be potentially explained by inconsistent patterns in AMF‐RSI within Glomeraceae. Therefore, we advocate for the distinguishing of AM fungal rhizophilic–edaphophilic strategies on a finer taxonomic level than previously performed. We believe that the AMF‐RSI provides such opportunity.

### Ecological applications

To characterize the traits of whole communities of AM fungi, we compared the community‐weighted AMF‐RSI to test the hypothesis that tillage decreases the share of edaphophilic AM fungi in soils. Since we were able to calculate AMF‐RSI for > 300 common AM fungal VTs, we were able to calculate CWM with reliable community coverage. CWMs, which are commonly used in the ecology of plants and other organisms (Diekmann, 2003; Violle *et al*., 2007; Mäkinen *et al*., 2025), combine species abundance and trait information to generate an average of ecologically relevant trait values in whole communities (Borgy *et al*., 2017).

Using this approach, we found that AM fungal communities under tillage were skewed toward taxa showing more rhizophilic strategy (Fig. 5). Numerous previous studies showed significant decreases in AM fungal richness due to tillage (Jansa *et al*., 2002; Verbruggen & Kiers, 2010; Brito *et al*., 2012; Säle *et al*., 2015). The detrimental effect of tillage on AM fungi was traditionally explained by physically breaking the hyphal connections in soil, which are essential for the colonization of new roots and for maintaining AM fungal populations. Our results indicate that AM fungal taxa, which preferentially associate with soil, are more sensitive to tillage disturbance. This analysis suggests that the AMF‐RSI has potential to better understand assembly rules of AM fungal communities. Similarly, the AMF‐RSI can be potentially used to better understand the role of different AM fungal taxa in ecosystem processes, such as soil carbon sequestration (Ao *et al*., 2025; Conti *et al*., 2025), soil aggregation, capacity to connect multiple plants, and form a ‘common mycorrhizal network’ (Alaux *et al*., 2021) or enable migration of rhizobia and other microbes over long distances to the roots (He *et al*., 2024). Alternatively, the NT management was associated with cultivation of cover crops, which could also potentially shift the AM fungal communities compared with the tilled soils. However, due to a high variability in vegetation composition of cover crops, it is likely that their effect on CWM of AMF‐RSI was rather neutral and the observed pattern can be attributed to the tillage itself.

### Conclusions

To conclude, this is a first study that provides quantitative trait representing the relative investment of AM fungal taxa into extra‐ vs intraradical structures. We calculated the AMF‐RSI for > 300 AM fungal taxa. We showed that preferential association with roots vs soil is phylogenetically conserved among AM fungi at the genus level rather than family level as indicated by previous research. We also showed that this trait can be used to better understand the assembly rules of AM fungal communities due to mechanical disturbance of the soil environment.

## Competing interests

None declared.

## Author contributions

PK and ZK coordinated this project. IO, FW, ZK and PK designed the study. FW, PB, FB, JS, TV, ZK and PK collected the data. KK and LM performed the field experiment. JS performed the pot experiment. IO, LBD, OO and PK developed the methodology. IO, FW, LBD and OO analyzed the data. PK wrote the original draft. IO, FW and ZK reviewed the first draft. All the authors reviewed and contributed to the final version of the manuscript. IO and FW contributed equally to this work.

## Disclaimer

The New Phytologist Foundation remains neutral with regard to jurisdictional claims in maps and in any institutional affiliations.

## Supporting information


**Fig. S1** Distribution of root (blue) and soil (red) samples from the GlobalAMFungi database and included in the 100‐km dataset (i.e. includes soil samples with a reference root sample within 100‐km distance and root samples with a reference soil sample within 100‐km distance).
**Fig. S2** Comparison of environmental conditions between root and soil samples of the 100‐km and 1000‐km datasets.
**Fig. S3** Mixing statistics for the Hierarchical Modeling of Species Community model with 1000 km dataset.
**Fig. S4** Mixing statistics for the Hierarchical Modeling of Species Community model with 100 km dataset.
**Fig. S5** Relationship between arbuscular mycorrhizal root–soil association index computed from the four datasets.
**Fig. S6** Comparison of model uncertainties measured as the posterior inter‐quartile ranges of arbuscular mycorrhizal root–soil association index across four datasets.
**Fig. S7** Correlation of log‐transformed arbuscular mycorrhizal root–soil association index with log‐transformed neutral lipid fatty acid root–soil ratio of eight studied arbuscular mycorrhizal fungal isolates.

**Table S1** Mean values, together with 70% and 90% credible intervals and categorical classification of arbuscular mycorrhizal fungal root–soil association index of 301 AM fungal Virtual Taxa, based on the 1000‐km distance and 97% sequence similarity dataset.
**Table S2** Mean values, together with 70% and 90% credible intervals and categorical classification of arbuscular mycorrhizal fungal root–soil association index of 268 AM fungal Virtual Taxa, based on the 100‐km distance and 97% sequence similarity dataset.
**Table S3** Mean values, together with 70% and 90% credible intervals and categorical classification of arbuscular mycorrhizal fungal root–soil association index of 211 AM fungal Virtual Taxa, based on the 1000‐km distance and 99% sequence similarity dataset.
**Table S4** Mean values, together with 70% and 90% credible intervals and categorical classification of arbuscular mycorrhizal fungal root–soil association index of 173 AM fungal Virtual Taxa, based on the 100‐km distance and 99% sequence similarity dataset.
Please note: Wiley is not responsible for the content or functionality of any Supporting Information supplied by the authors. Any queries (other than missing material) should be directed to the *New Phytologist* Central Office.

## Data Availability

Sequence data generated during the current study are deposited in SRA (PRJNA1377471). Sufficient data and R scripts to reproduce the analyses of this study are accessible via Zenodo repository at doi: 10.5281/zenodo.18325103.
