# Supplementary material for: A root–soil association index reveals life‐history strategies of arbuscular mycorrhizal fungi
Source: New Phytol. 2026 Jun 4;251(5):2932–47. doi: 10.1111/nph.71338 (PMC13443022; doi:10.1111/nph.71338)
Supplement: Supplementary file 1 [file NPH-251-2932-s001.pdf]

New Phytologist Supporting Information

Article title: **A root-soil association index reveals life-history strategies of arbuscular mycorrhizal fungi**

Authors: Iñaki Odriozola[1,a], Felix Wesener[1,a], Petr Baldrian[1], Florian Barbi[1], Lukas Bell-Dereske[1], Karel Klem[2], Lenka Meszárošová[1], Otso Ovaskainen[3], Jakub Skřivánek[1,4,5], Tomáš Větrovský[1], Zuzana Kolaříková[5] and *Petr Kohout[1,4]

Article acceptance date: 26 May 2026

1) Institute of Microbiology of the Czech Academy of Sciences, Vídeňská 1083, 142 00 Prague, Czechia
2) Global Change Research Institute of the Czech Academy of Sciences, Bělidla 986/4a, 603 00 Brno, Czechia
3) Department of Biological and Environmental Science, University of Jyväskylä, P.O. Box 35, FI-40014 Jyväskylä, Finland
4) Faculty of Science, Charles University, Albertov 6, 128 00 Prague, Czechia
5) Institute of Botany of the Czech Academy of Sciences, Zámek 1, 252 43 Průhonice, Czechia
a) These authors contributed equally to this work.

*corresponding author:
Petr Kohout
Faculty of Science,
Charles University
Albertov 6, 128 00 Prague, Czechia
petr.kohout@natur.cuni.cz

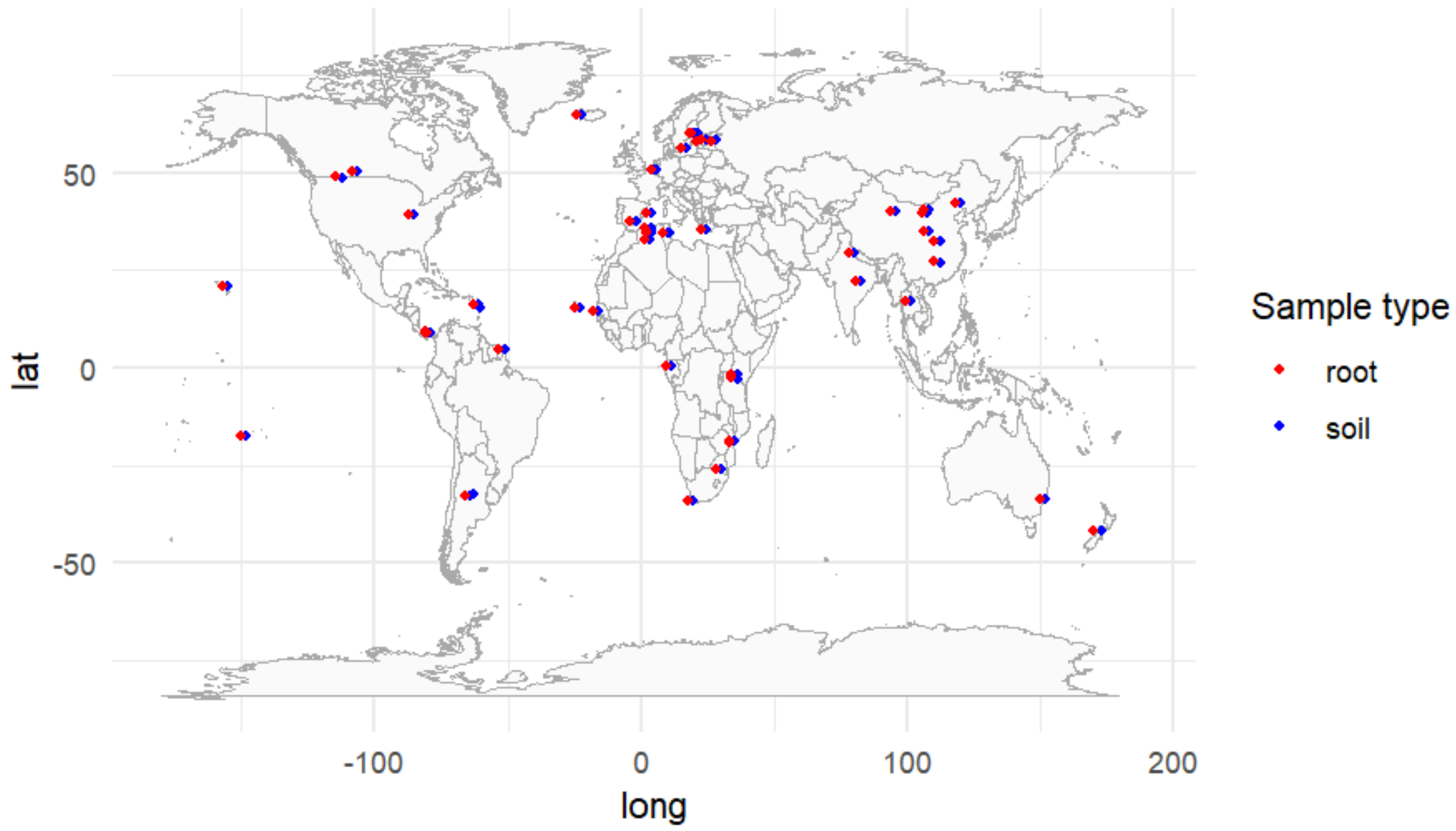

Fig. S1. Distribution of root (blue) and soil (red) samples from the GlobalAMFungi database and included in the 100 km dataset (i.e. includes soil samples with a reference root sample within 100 km distance and root samples with a reference soil sample within 100 km distance). This data was used for modelling of preferential root-soil association of arbuscular mycorrhizal fungal taxa. Data points were jittered along longitude by adding 1° to soil sample coordinates and subtracting 1° from root sample coordinates to improve visual distinguishability on the map.

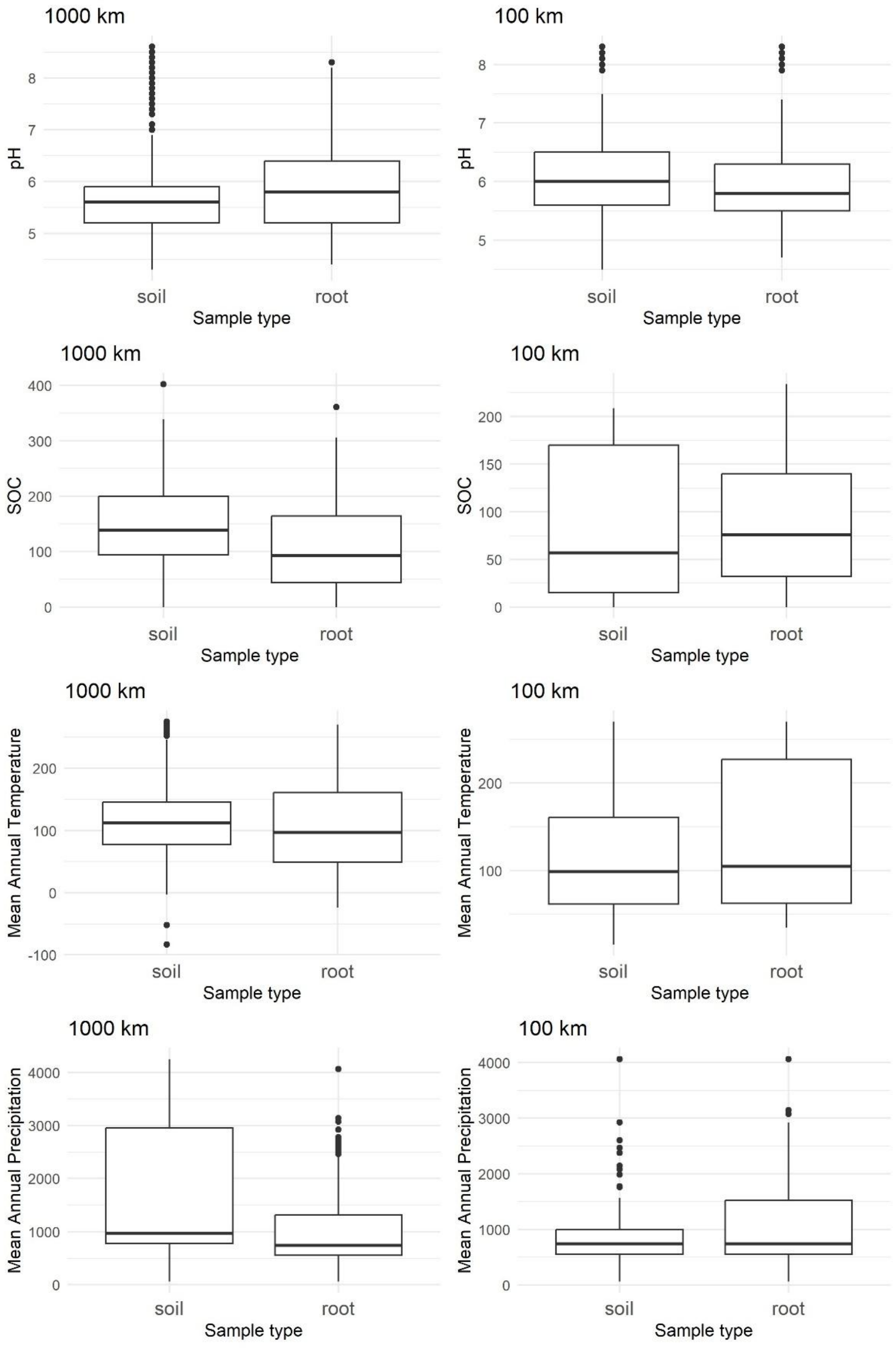

Fig. S2. Comparison of environmental conditions between root and soil samples of the 100 km and 1000 km datasets.

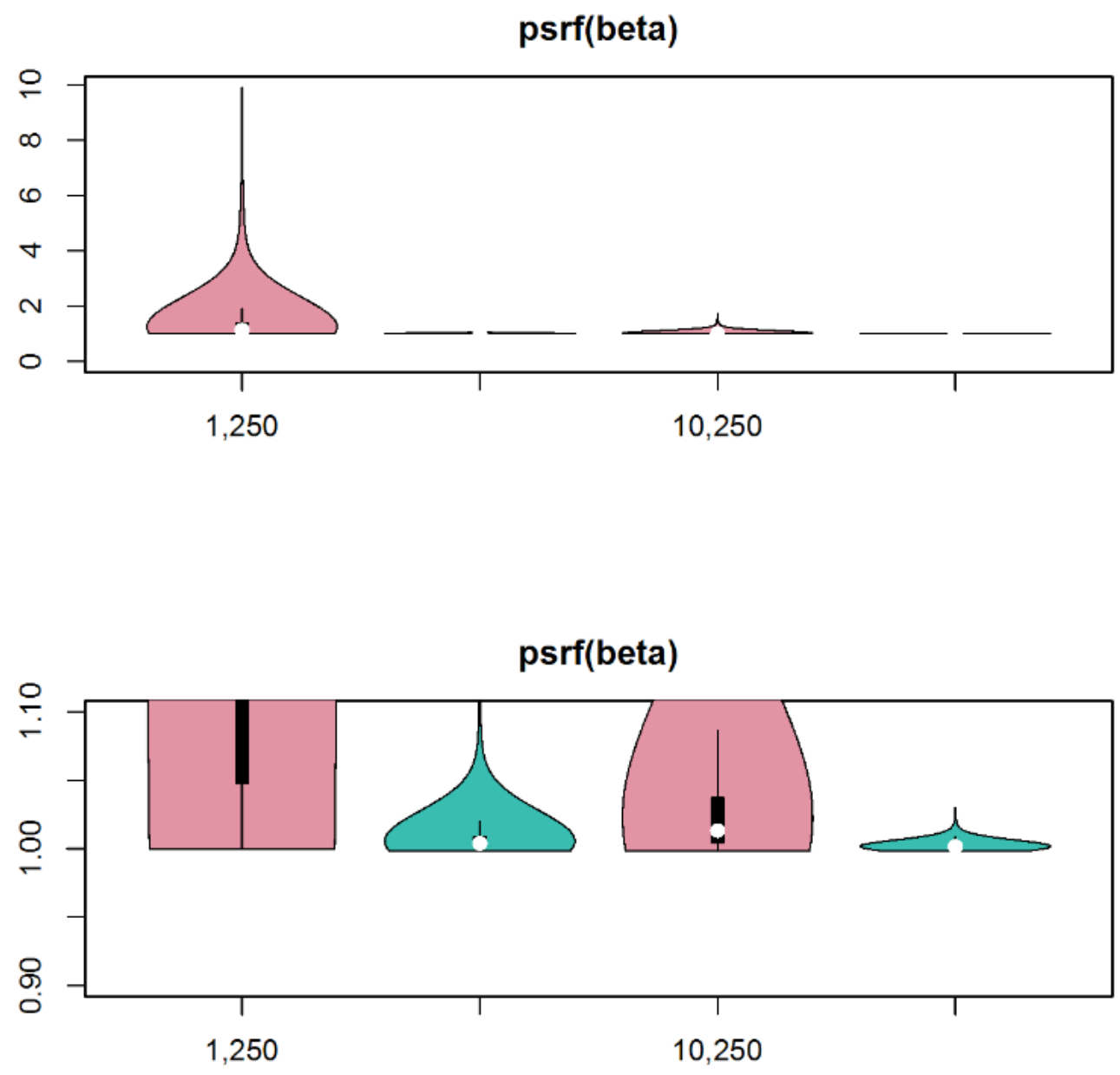

Fig. S3. Mixing statistics for the Hmsc model with 1000km dataset. Top panel shows the entire range for y axis whereas bottom panel focuses in the range 0.9-1.1. Potential Scale Reduction Factors close to one indicate satisfactory model convergence. Pink boxes are for presence absence submodels and green boxes are for abundance conditional-on-presence submodels. 1,250 indicates a thinning value of one and 250 posterior samples per chain; 10,250 indicates indicates a thinning value of 10 and 250 posterior samples per chain.

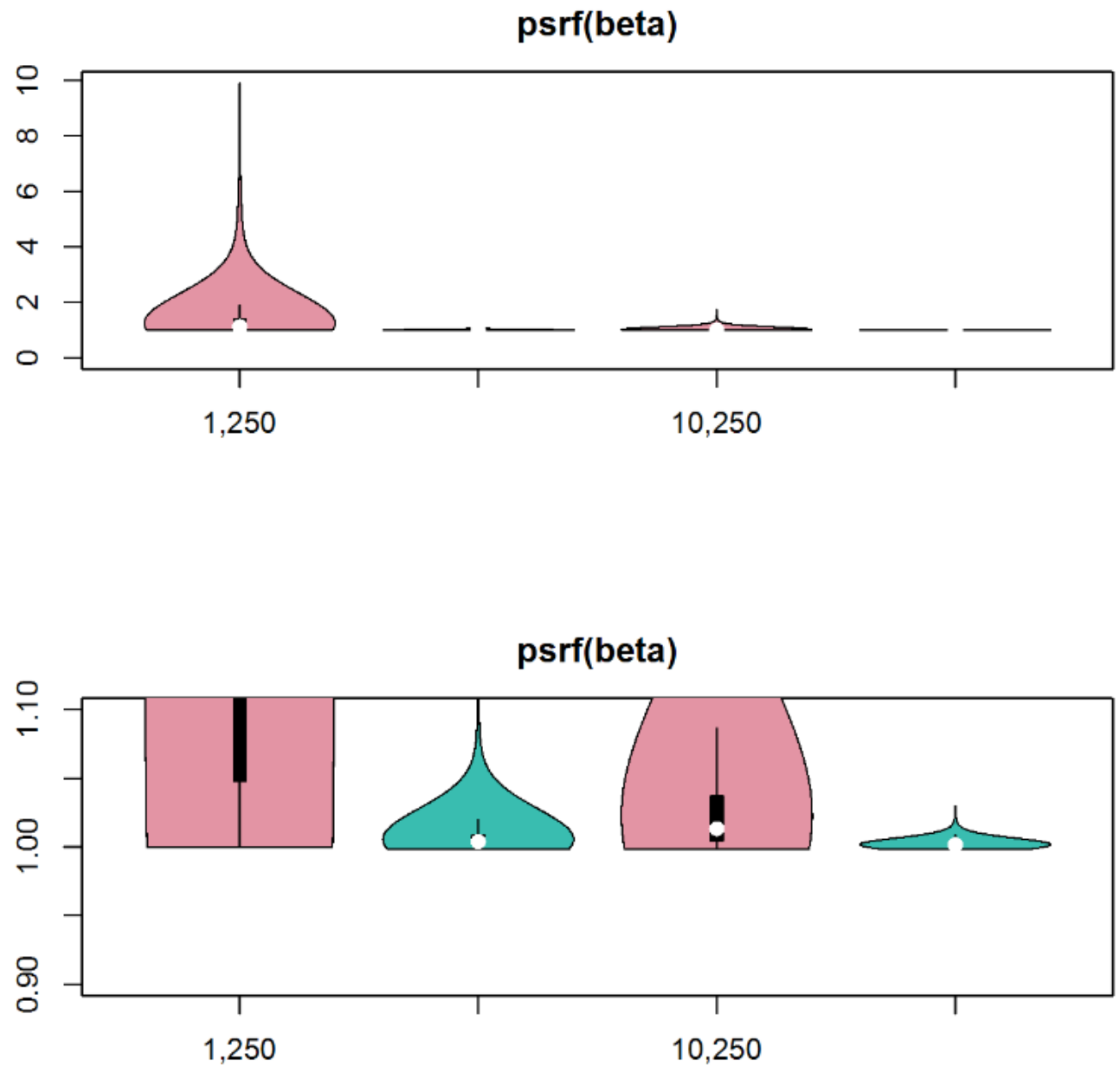

Fig. S4. Mixing statistics for the Hmsc model with 100km dataset. Top panel shows the entire range for y axis whereas bottom panel focuses in the range 0.9-1.1. Potential Scale Reduction Factors close to one indicate satisfactory model convergence. Pink boxes are for presence absence submodels and green boxes are for abundance conditional-on-presence submodels. 1,250 indicates a thinning value of one and 250 posterior samples per chain; 10,250 indicates indicates a thinning value of 10 and 250 posterior samples per chain.

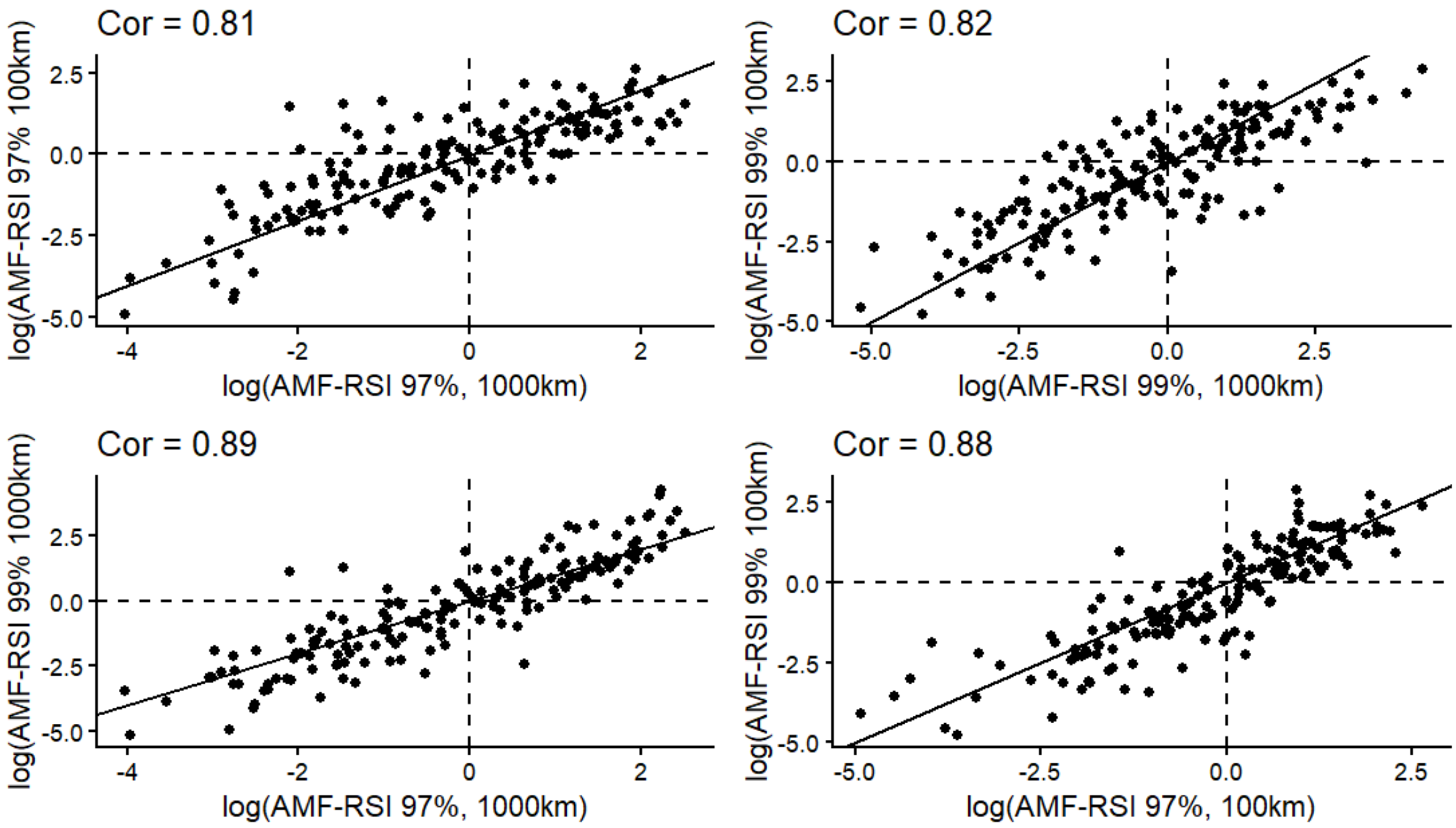

Fig. S5. Relationship between AMF-RSI index computed from the four datasets: 1000 km and 97% similarity; 100 km and 97% similarity; 1000 km and 99% similarity; 100 km and 99% similarity. The results are overall consistent: the few taxa with shifted sign in the index between datasets have log-ratios close to zero. Dashed lines depict log(AMF-RSI) values of zero, indicating no association with any of the substrates. The solid regression lines correspond 0 intercept and 1 slope line, indicating a 1:1 relationship between variables.

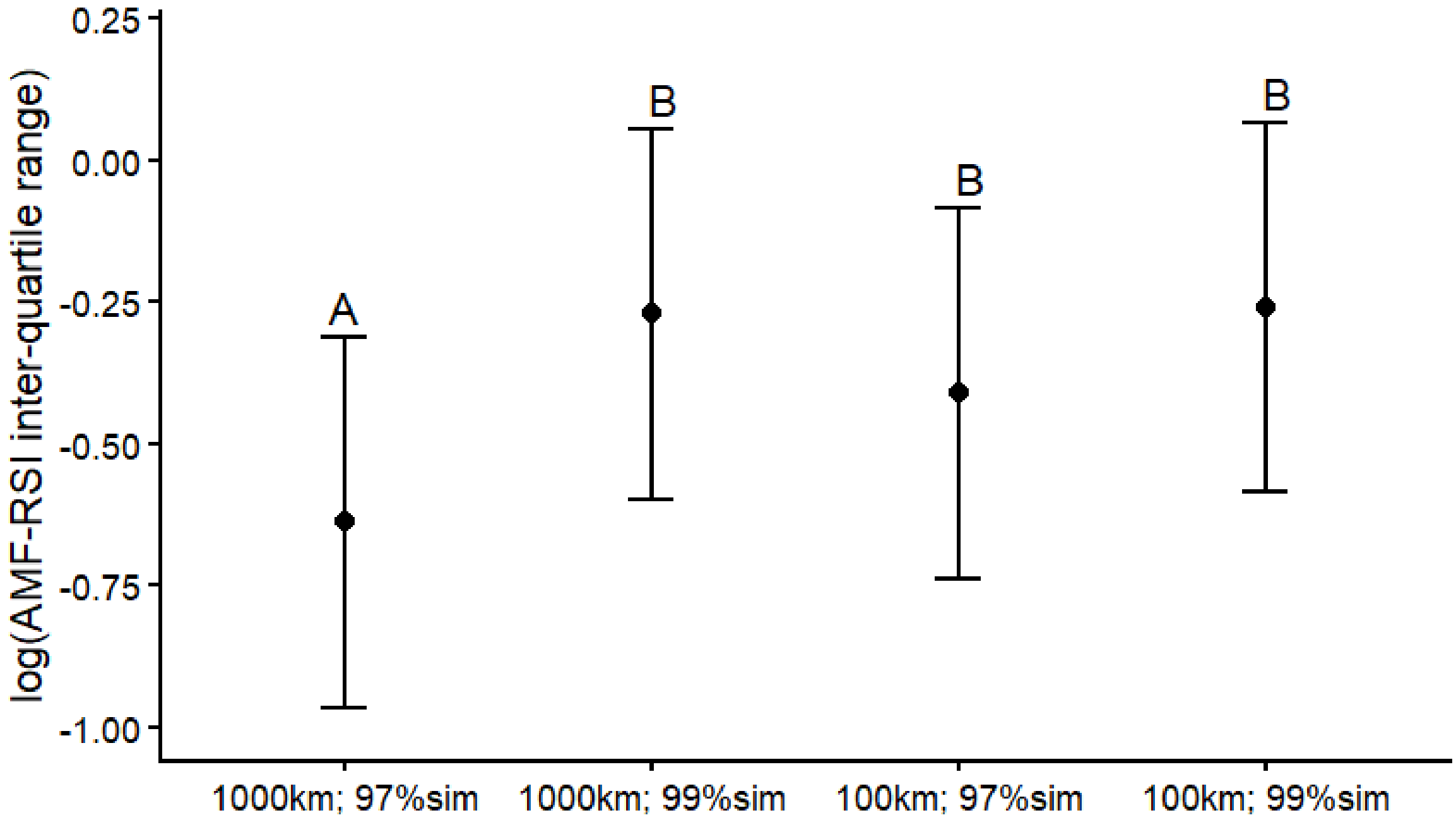

Fig. S6. Comparison of model uncertainties measured as the posterior inter-quartile ranges of AMF-RSI index across four datasets: 1000 km and 97% similarity; 100 km and 97% similarity; 1000 km and 99% similarity; 100 km and 99% similarity. 1000 km and 97% similarity dataset yielded posterior predictions with lowest uncertainty. Error bars depict Sidak's 95% confidence intervals. Letters above the boxes show Sidak pairwise tests of a linear mixed effect model with dataset (categorical variable with four levels) as fixed effect and species identity as random effect.

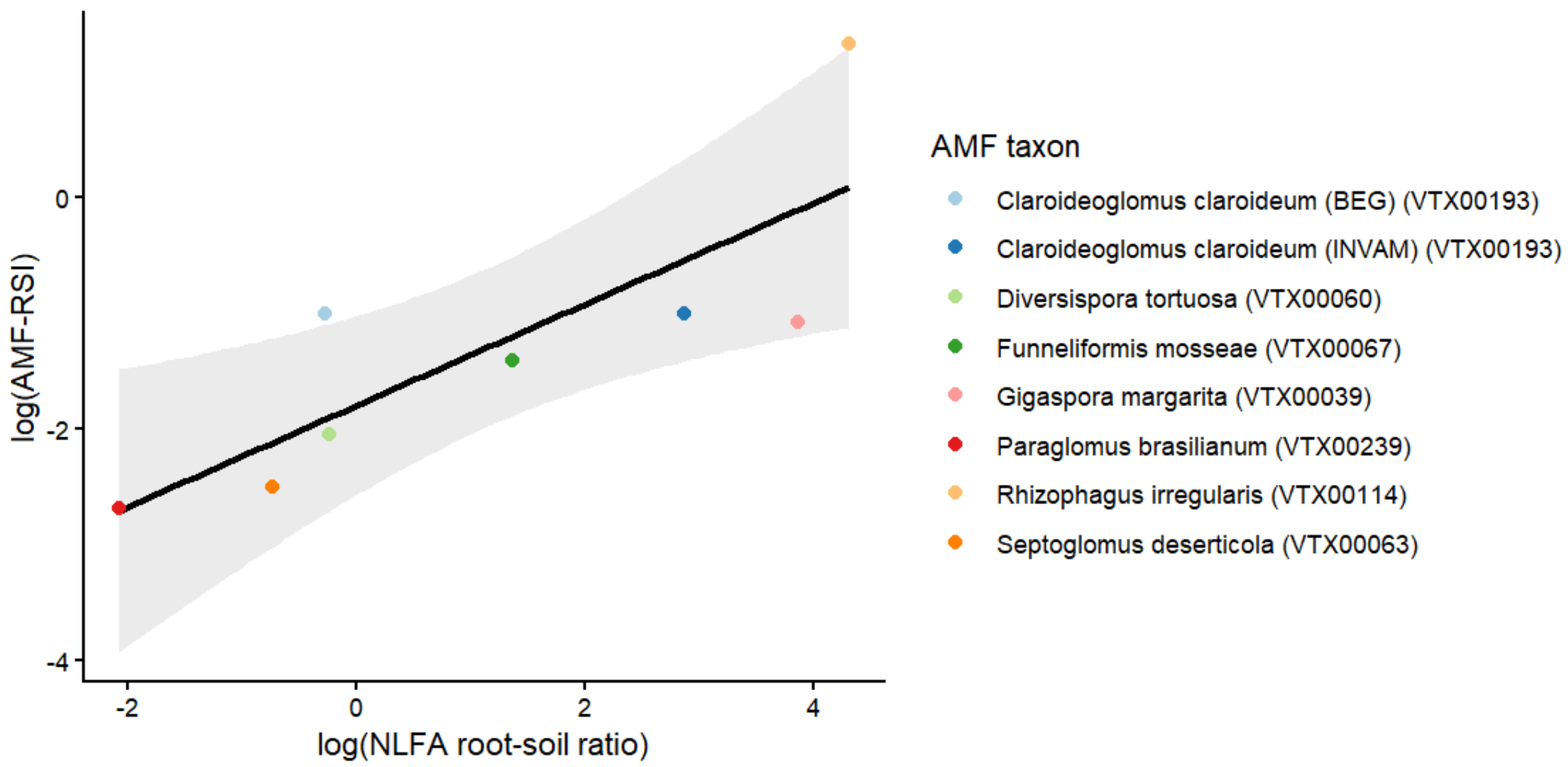

Fig. S7. Correlation of log-transformed AMF-RSI with log-transformed NLFA root-soil ratio of eight studied AM fungal isolates. NLFA root-soil ratio was calculated as a mean value of root-soil ratio of six replicates and then log transformed. AMF-RSI was derived from the corresponding AM fungal VT.
